# In Utero Exposure to Gestational Diabetes Alters DNA Methylation and Gene Expression of *CDKN2A/B* in Langerhans Islets of Rat Offspring 

**DOI:** 10.22074/cellj.2020.6699

**Published:** 2019-10-14

**Authors:** Zahra Nazari, Alireza Shahryari, Soraya Ghafari, Mohammad Nabiuni, Mohammad Jafar Golalipour

**Affiliations:** 1. Department of Biology, Faculty of Sciences, Golestan University, Gorgan, Iran; 2. Stem Cell Research Center, Faculty of Medicine, Golestan University of Medical Sciences, Gorgan, Iran; 3. Congenital Malformations Research Center, Golestan University of Medical Sciences, Gorgan, Iran; 4. Department of Cell and Molecular Biology, Faculty of Biological Sciences, Kharazmi University, Tehran, Iran

**Keywords:** DNA Methylation, Gestational Diabetes, Islets of Langerhans

## Abstract

**Objective:**

DNA methylation, a major epigenetic reprogramming mechanism, contributes to the increased prevalence of type 2 diabetes mellitus (T2DM). Based on genome-wide association studies, polymorphisms in *CDKN2A/B* are associated with T2DM. Our previous studies showed that gestational diabetes mellitus (GDM) causes apoptosis in β-cells, leading to a reduction in their number in pancreatic tissue of GDM-exposed adult rat offspring. The aim of this study was to examine the impact of intrauterine exposure to GDM on DNA methylation, mRNA transcription, as well as protein expression of these factors in the pancreatic islets of Wistar rat offspring. Our hypothesis was that the morphological changes seen in our previous study might have been caused by aberrant methylation and expression of *CDKN2A/B*.

**Materials and Methods:**

In this experimental study, we delineated DNA methylation patterns, mRNA transcription and protein expression level of *CDKN2A/B* in the pancreatic islets of 15-week-old rat offspring of streptozotocin-induced GDM dams. We performed bisulfite sequencing to determine the DNA methylation patterns of CpGs in candidate promoter regions of *CDKN2A/B*. Furthermore, we compared the levels of mRNA transcripts as well as the cell cycle inhibitory proteins P15 and P16 in two groups by qPCR and western blotting, respectively.

**Results:**

Our results demonstrated that hypomethylation of CpG sites in the vicinity of CDKN2A and CDKN2B genes is positively related to increased levels of *CDKN2A/B* mRNA and protein in islets of Langerhans in the GDM offspring. The average percentage of CDKN2A promoter methylation was significantly lower in GDM group compared to the controls (P<0.01).

**Conclusion:**

We postulate that GDM is likely to exert its adverse effects on pancreatic β-cells of offspring through hypomethylation of the *CDKN2A/B* promoter. Abnormal methylation of these genes may have a link with β-cell dysfunction and diabetes. These data potentially lead to a novel approach to the treatment of T2DM.

## Introduction

Gestational diabetes mellitus (GDM) is generally deﬁned as hyperglycemia that is ﬁrst recognized during pregnancy. GDM affects up to one in seven pregnancies worldwide (1). It is often associated with further pregnancy complications like preeclampsia or preterm delivery. Additionally, about 50% of the female patients suffering from GDM are likely to develop overt diabetes (2). In Addition to having adverse consequences for mothers, GDM is also associated with a high vulnerability to facing short-term detrimental after-effects such as macrosomia, neonatal hypoglycemia and neonatal cardiac dysfunction, as well as long-term difficulties including lifelong obesity and type 2 diabetes mellitus (T2DM) in the offspring (3-5). It has been demonstrated that animal offspring of diabetic mothers overtly develop diabetes later in life (6-8). A study carried out in the US has shown that 47.2% of diabetic cases among younger people could be attributed to diabetes or obesity of their mothers during her pregnancy (9). In animal models of GDM, a decline in insulin secretion as well as β-cell impairment has been observed in mature offspring (6, 7). Our group previously investigated the effect of GDM on morphological and histological features of pancreas in adult rat offspring. We demonstrated that GDM causes a significant reduction in β-cell mass, islet number and islet diameter in adult rat offspring (8). Our previous data also revealed that in rats, offspring of GDM mothers have more apoptotic β-cells compared to the controls (10).

Currently, little is known about how GDM-exposure contributes to the susceptibility to diabetes development in the offspring. Many research studies link the cell cycle regulators like cyclin-dependent kinase 4 (*Cdk4*) and retinoblastoma protein (*pRB*) to the risk of developing diabetes. *CDK4-pRB-E2F1* pathway has direct effects on proliferation and insulin secretory capacity of β-cells (10-13). In our previous study on the offspring of GDM rats, we showed that GDM downregulates CDK4-pRBE2F1 pathway in Langerhans islets (14). Proteins of the INK4 family, like *CDKN2A* and *CDKN2B* (encoding the cell cycle inhibitory proteins p16^INK4b^ and p15^INK4a^, respectively), can negatively regulate the activity of Cdk4 and subsequently block cell cycle progression. This indicates that upregulation of *CDKN2A/CDKN2B* genes may restrict the *CDK4-pRB-E2F1* pathway as well as β-cell proliferation. Other studies have identiﬁed novel T2DM susceptibility loci within the *CDKN2A/B* gene regions (chromosome 9p21 in humans and chromosome 5q32 in rats) (15, 16).

Current studies link DNA methylation with both type 1 and type 2 diabetes, at least partially through changes in the β-cell proliferation (17-20). Recent works have however shifted the focus to the effects of intrauterine exposure to hyperglycemia on DNA methylation of genes important in β-cell proliferation and function, which can increase the risk of diabetes in the offspring (21, 22).

In the same line as our previous studies on the effects of GDM on histological, morphological and molecular aspects of pancreas in the offspring (8, 9, 12), in the present study we evaluated the impact of streptozotocin (STZ)-induced GDM on DNA methylation and gene expression of *CDKN2A/B* in pancreatic islets of adult offspring in Wistar rats. We postulated that intrauterine hyperglycemic environment affects the β-cells of the offspring by the loss of *CDKN2A/B* methylation. We performed targeted-bisulfite sequencing to evaluate the CpG islands methylation levels in the regulatory regions of *CDKN2A* and *CDKN2B* in the pancreatic islets of the offspring. In addition, mRNA expression of *CDKN2A/B* and protein levels of p15 and p16 (proteins encoded by *CDKN2B* and *CDKN2A*, respectively) were analyzed by qPCR and western blotting.

## Materials and Methods

### Animals

This research was an experimental study, in which a total of 40 female and 15 male Wistar rats with an average age of 10-12 weeks were utilized. The animals were obtained from Golestan University of Medical Sciences, Gorgan, Iran. All animal procedures presented in this study adhered to the guidelines proposed by the Institutional Animal Care and Use Committee at Golestan University of Medical Sciences, Gorgan, Iran (code: IR.GOUMS. REC.1394.247).

### Induction of experimental gestational diabetes mellitus

As many as 14 female rats were independently paired with a male rat for the purpose of breeding. Following copulation, observation of vaginal plaques was considered as the day zero of gestation. Then, they were equally randomized into control and GDM group. Animals in the GDM group received a single dose of STZ solution through intraperitoneal injection (40 mg/kg bw prepared freshly in citrate buffer, 0.1 mol/L) on day zero of gestation, while the control group received a similar volume of citrate buffer only. We administrated STZ on day zero of gestation because its administration before pregnancy has adverse effects on mating behavior. On the other hand, STZ has a half-life of about five minutes, so it is unlikely that exposure to STZ on day zero affects the earliest stages of embryogenesis. 72 hours after STZ administration, tail incision method was used to measure fasting blood glucose level using a glucometer (ACCU-CHEK Glucometer, Roche Diagnostics). Rats with high serum glucose levels in the range of 120-250 mg/dl were chosen and considered as diabetic models (23). Following spontaneous delivery, pups were allowed to mature for 15 weeks. As for investigation of the effect of GDM during embryonic period (not the effect of breastfeeding by diabetic mothers) on pancreas development, all control and offspring of gestational diabetes (OGD) infants were milked by normal mothers. OGD and control groups were sacrificed and their pancreatic tissues were collected and processed for isolation of islets of Langerhans.

### Islets of Langerhans isolation

Collagenase digestion technique was used for isolating pancreatic islets from the control and OGD groups (24). In summary, cannula was inserted into the common bile duct. Digestion solution, containing 2.0 ml of 0.2 mg/ liberase TL or liberase Thermolysin Low (Roche, USA) and 10 pg/ml DNase (Takara, Japan) in serum-free Roswell Park Memorial Institute1640 medium (RPMI 1640 medium, Invitrogen, Germany), was injected into the cannula. Pancreas were placed in a 1.5 ml microtube and incubated at 37˚C for 15 minutes. The tubes were later filled with 10 ml of RPMI 1640 containing 10% fetal bovine serum (FBS, Invitrogen, Germany) serum and were kept on ice for 5 minutes to allow for enzyme deactivation. Following a phase of centrifugation at 800 RPM for 2 minutes, the islets of Langerhans were isolated through centrifugation on a Ficoll gradient (Sigma-Aldrich, USA). The islets were then gathered from the histopaque/media interface and passed through a 100-μm cell strainer (BD Falcon). Finally, the pancreatic islets were rinsed and stored at -80˚C until further extractions. The above procedure was repeated for each animal.

### RNA, DNA and protein extractions from islets of langerhans

By applying the total RNA purification kit (Jena Bioscience, Germany), total RNA was elicited from the pancreatic islets. All islets from 5 rats were pooled to create a uniform sample for different extractions. DNA was isolated using the NucleoSpin Tissue XS kit (MN, Germany). Both DNA/RNA quantity and purity were calculated by NanoDrop ND-1000 spectrophotometer, while RNA integrity was backed up by showing the intact 28s and 18s bands on gel electrophoresis using 1% agarose gels. Furthermore, total protein from pancreatic islets of the control and GDM offspring were obtained using a total protein extraction kit as specified by the manufacturer’s directions (Merck Millipore, Germany). The Pierce BCA protein assay kit (Thermo Fisher Scientific) was used to measure protein concentration from total cell lysates.

### Bisulfite-specific polymerase chain reaction and Sanger sequencing

Using the Zymo EZ DNA Methylation Gold Kit (Zymo Research, USA), bisulfite treatment was added to 500 ng of genomic DNA, as instructed by the manufacturer. Primers for detecting the methylation pattern of the *CDKN2A* and *CDKN2B* promoters, which are listed in Table 1, were designed by Bisulfite Primer Seeker (Zymo Research, USA) software. Eighteen CpG sites, located between -161 and +281 bp of the CDKN2A promoter and 39 CpG sites, located between -109 and +285 bp of the *CDKN2B* promoter were investigated with specific primers. Amplification of bisulfite converted DNA was performed using EpiTaq HS kit (for bisulfite-treated DNA, Takara, Japan). The thermal cycling phases included a preparatory denaturation at 98 ˚C lasting for 10 seconds as well as a two-step amplification program of 35 cycles at 55 ˚C and 72 ˚C each for 30 seconds. Bisulfite-amplified PCR products were refined by taking advantage of a AccuPrep PCR Purification Kit (Bioneer) and were later directly sequenced using an automatic sequencer (ABI PRISM-77). We derived two DNA sequence per animal for a total of n=6 sequenced samples for OGD and controls. The aligned reads and levels of methylation in both OGD and control groups were visualized using the pairwise sequence alignment online software (https://www.ebi.ac.uk/Tools/psa/).

### Quantitative polymerase chain reaction

Islets of Langerhans RNA samples were reversetranscribed using prime script RT reagent kit (Takara, Japan). Primers for respective genes were designed using the PerlPrimer software (Bio-Rad, USA) and synthesized by the Metabion Company (http://www.metabion. com). The oligonucleotide sequences of primers utilized for qPCR are presented in Table 1. The quantitative polymerase chain reaction (qPCR) was carried out in the Applied Biosystems 7300 Real-Time PCR System (Life Technologies, USA) with the SYBR-Green PCR Master Mix kit (Takara, Japan). We used beta-actin as the housekeeping gene and cDNA from offspring islets of the control group as calibrator. The expression level for each sample was measured using the cycle threshold (Ct) value while relative mRNA expression was calculated using the 2^-ΔΔCt^ formula. All real-time PCR experiments were conducted in triplicates.

### Western blot analysis

An immunoblot assay was evaluated for the effect of GDM on p15 and p16 protein expression in the pancreatic islets of the offspring. In short, 35 μg of the total proteins from each control and OGD groups were run on 10% polyacrylamide gels and transferred to nitrocellulose membranes sheets using a transblot system (Bio-Rad, USA). Western blot analysis was performed using the p15 (Sigma, USA) and p16 (Proteintech, Japan) primary antibodies. Monoclonal GAPDH antibody was used as a loading control (Santa Cruz Biotechnology, Japan). Immune-blot assay kit (Bio-Rad, USA) was used to visualize the protein bands. After scanning of the blots, they were quantified using Quantity One Software (BioRad, USA).

**Table 1:** Bisulfite sequencing polymerase chain reaction (PCR) and qPCR primer name, sequences, product size and number of CpGs


Gene	Primer sequence (5ˊ-3ˊ)	Product size (bp)	Number of CPGs

CDKN2A (Genomic)	F: GGGGTGTGGAATTAGGTTAGGAGTAAAATGTG	342	18
	R: TTCACTCTTCTTAAACAAAAATTATCTCACTAC		
CDKN2B (Genomic)	F: TTTATTATAGTTGTTGGGTTTTTAGAGAGGAG	394	39
	R: ATTTTTACCCTTACAAAAAACAAAACCTACCTCCC		
CDKN2A (mRNA)	F: CTCTGCAGATAGACTAGCCA	127	-
	R: CATCATCACCTGTATCGGG		
CDKN2B (mRNA)	F: AGATCCCAACGCCGTCAAC	184	-
	R: CAGCACCATTAGCGTGTCCAG		
β-actin (mRNA)	F: AAGATCAAGATCATTGCTCCTC	169	-
	R: CTCAGTAACAGTCCGCCT		


### Data analysis

For qPCR, bisulfite sequencing and western blot analysis in the 15-week-old offspring, 6 females and 6 males from 6 litters, were studied for each group. Also, we used 6 blood samples of each control and diabetic pregnant rat to measure its fasting blood glucose level. Statistical analyses of all data were performed using the SPSS 18.0 statistical analysis software (SPSS Inc., Chicago, IL, USA) and data were expressed as the mean ± standard deviation (SD). One-way ANOVA was used to determine significant differences in all the parameters (blood glucose level, mRNA expression, DNA methylation and protein level) between the control and OGD groups. For promotor methylation data analysis, we compared the average methylation of CpGs in the control and OGD groups. Significance was determined at P<0.05.

## Results

### Blood glucose level

**Fig 1:**
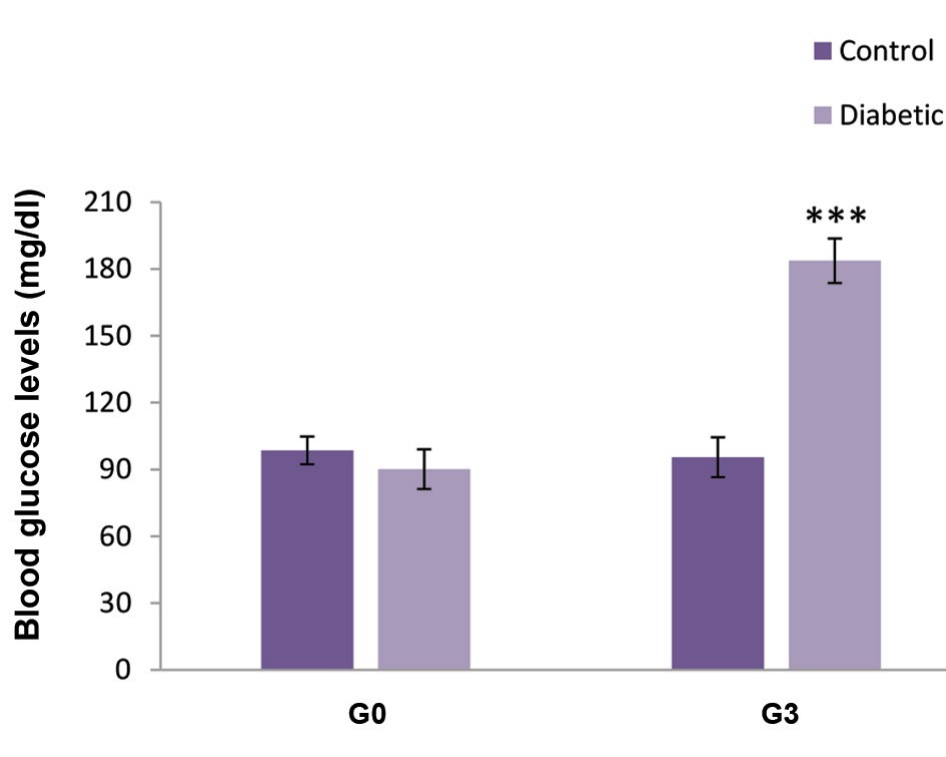
Blood glucose concentrations in diabetic and control pregnant rats. G0; Day 0 of gestation and G3; Day 3 of gestation. All values are presented as means ± SEM. ***; P<0.001, n=12.

A significant increase was observed in the fasting blood glucose levels of STZ-induced GDM rats (Fig .1). Seventy-two hours after STZ injection, nearly 70% of dams developed hyperglycemia (P<0.001).

### Bisulfite DNA sequencing

Bisulfite DNA sequencing was employed to identify the methylation levels of the CpGs in the CDKN2A and CDKN2B promoters. Six OGD and six control DNA samples were amplified with a 342 bp fragment in CDKN2A promoter, which comprised of 18 CpG sites and a 394 bp fragment in the CDKN2B promoter, containing 39 CpG sites.

**Fig 2:**
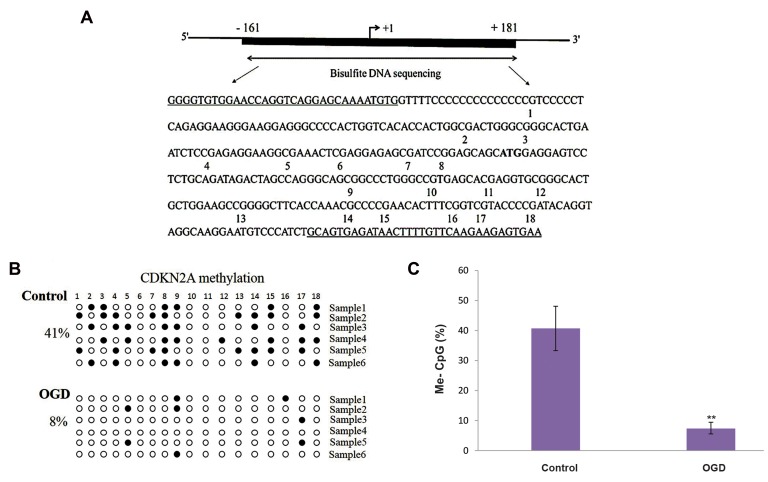
Bisulfite DNA sequencing of the CpGs in the *CDKN2A* promoter. A. Diagrammatic illustration of the *CDKN2A* promoter-associated CpG islands, which cover the region from -161 to +181 (the translation initiation site ATG as +1). The region analyzed by bisulfite sequencing polymerase chain reaction (PCR) is depicted. This region covers 342 bp and consists of 18 CpG, B. Methylation patterns of the *CDKN2A* CpG island in six control and six OGD samples (each line represents an independent sample). Methylated and unmethylated CpG sites are represented as solid and open circles, respectively, and C. Statistical analysis of methylation in the two groups. Data are presented as mean ± SD. **; P<0.01, compared with the controls.

**Fig 3:**
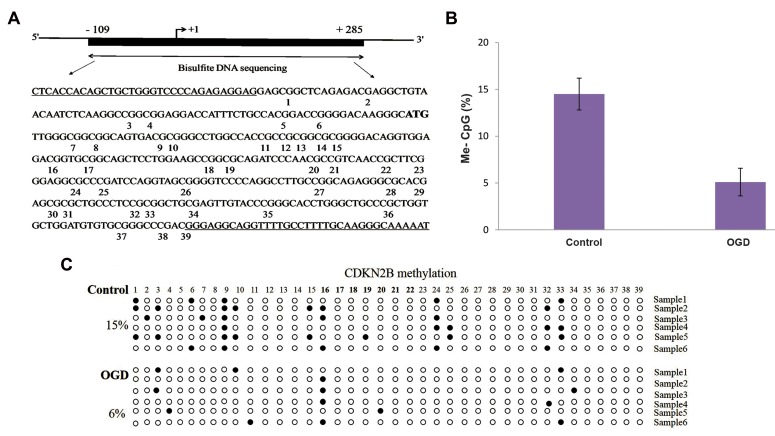
The CpG island in the *CDKN2B* promoter showed by bisulfite sequencing. **A.** Illustrative sketch of the CDKN2B promoter, which covers the region from -109 to +285. The region analyzed by bisulfite sequencing polymerase chain reaction (PCR) is depicted. This region covers 394 bp and includs 39 CpG dinucleotides, **B.** Methylation patterns of the *CDKN2B* CpG island in six control and six OGD samples. Each circle represents a single CpGs (closed and open circles show methylated and unmethylated regions, respectively), and **C.** Statistical analysis of methylation in the two groups. Data are presented as mean ± SD, P=0.064.

**Fig 4:**
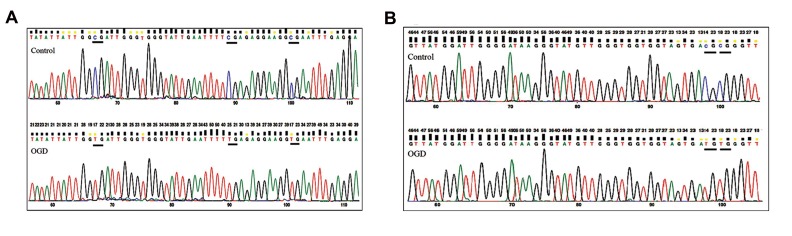
The same sections of the sequencing trace for **A.** *CDKN2A* and **B.** *CDKN2B* in the control and offspring of gestational diabetes (OGD) samples are presented. Underline sections indicate methylated-CpG cytosines (blue) in the control samples that are not converted and the same CpG cytosines in the OGD samples that are unmethylated and converted to thymines (red). CpG2, CpG4 and CpG5 for CDKN2A and CpG9 and CpG10 for *CDKN2B* are methylated in the control samples while they are unmethylated in OGDs.

Sequencing data confirmed methylation in CpG islands of the control and OGD samples, while the overall methylation patterns were distinct. Our data revealed that more CpG islets were methylated in the samples derived from controls than the ones acquired from OGDs. We found that the CDKN2A promoter (nucleotides-161 to +181 bp) (Fig .2A) was more methylated in the control samples (40.7%) than in the OGDs (7.3%) and the differences between them were significant (P<0.01) (Fig .2B, C). Also, bisulfite sequencing of the 394bp region of the CDKN2B promoter (nucleotides-109 to +285 bp) (Fig .3A) in OGD samples, determined the hypomethylation of CpG islands in this region. As shown in Figures 3B and C, the average DNA methylation in the control and OGD samples were 14.5% and 6%, respectively; thought this difference was not significant. Figure 4 demonstrates a section of bisulfite genomic sequencing chromatography for CDKN2A and CDKN2B. The sequenced region and methylation pattern for CDKN2A and CDKN2B are depict in Figure 2A, B and Figure 3A, B respectively. Interestingly, the control samples shared some common methylation sites (CpG8 for CDKN2A and CpG9 for CDKN2B) while these CpGs were all unmethylated in our OGD samples (Fig .2B, Fig .3B).

### Analysis of mRNA expression

**Fig 5:**
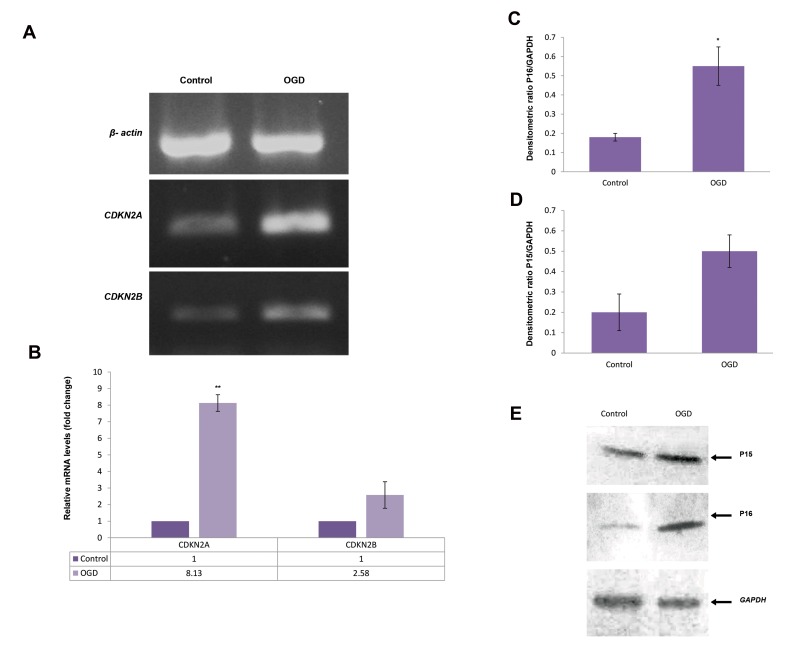
Real-time polymerase chain reaction (PCR) and western blotting analysis of CDKN2A/B in the pancreatic islets of the control and offspring of gestational diabetes (OGD) rats. *CDKN2A/B* mRNAs were determined by quantitive PCR (qPCR). **A.** Reverse transcription polymerase chain reaction (RT-PCR) analysis for the mRNA expression of *CDKN2A/B*, **B.** Real-time PCR analysis for the mRNA level of *CDKN2A/B* in control and OGD samples, **C, D.** Densitometric quantiﬁcation of western blotting bands for P15 and P16 proteins in the two groups, and **E.** Nitrocellulose blot of sodium dodecyl sulfate polyacrylamide gel electrophoresis SDS-PAGE gel developed with immune-blot assay kit, column 1, control group; column 2, OGD group. *GAPDH* was used as the housekeeping gene. Data are presented as means ± SD, and the experiments were repeated independently three times. *; P<0.05 and **; P<0.01.

After bisulfite sequencing analysis of *CDKN2A/B* genes, we inspected the correlation between DNA methylation and mRNA levels in the pancreatic islets of the control and GDM offspring. *CDKN2A* and *CDKN2B* mRNA expression was detected in both groups, but their expression was significantly upregulated in the OGD samples, which was correlated with hypomethylation in the promoter region of these genes. The strongest correlation between *CDKN2A* mRNA levels and the methylation levels of the CpG islets was detected at -161 to +181 region. Our result suggested that the levels of *CDKN2A* methylation were significantly higher in OGD samples compared to the controls (P<0.01, Fig .5A, B). Furthermore, *CDKN2B* mRNA levels were higher in the OGD group compared to the controls; although the difference was not statistically significant (Fig .5A, B).

### Western blot analysis results

Western blotting of Langerhans islets protein samples from OGD and control groups, identiﬁed two slim bands at 15 and 16kDa for P15 (CDKN2B) and P16 (CDKN2A) proteins, respectively. Both proteins showed an increased band intensity in the OGD samples (Fig .5C-E), which correlates with and confirms our results observed in the real-time PCR analysis. Quantitative analysis of western blotting bands showed that gestational diabetes causes a significant increase in the expression level of P16 protein in the islets of of fspring (P<.05, Fig .5,C-E).

## Discussion

In the present study, we have analyzed DNA methylation levels of the *CDKN2A/B* genes in pancreatic islets of OGD rats. Data indicated that decreased DNA methylation levels at CpG sites in the *CDKN2A* and *CDKN2B* gene promoter in pancreatic islets of the rat offspring were associated with maternal hyperglycemia. Furthermore, p15 and p16 mRNA and protein levels in the OGD samples increased as compared to the control group, which is probably due to promoter hypomethylation of these genes.

Various animal studies suggest that following gestational diabetes, the offspring exhibit systemic insulin resistance as well as elevated circulating insulin and glucose compared to the offspring of normal dams (6-8). Recently, our team investigated the effects of GDM on some histological aspects of the pancreas in adult rats’ offspring (8, 9). Gomori staining showed that β-cell number, islet number and islet diameter is significantly reduced in the offspring of diabetic mothers (8). Also, a separate study by our group indicated that the number of apoptotic β-cells grows in OGDs. In addition, we observed that adult offspring rats mainly developed mild hyperglycemia (9). Strong evidence exists that maternal hyperglycaemia increases the risk of insulin resistance, obesity, and type 2 diabetes in young adult offspring. Although much of the molecular pathways, through which GDM mediates its effects remain unknown. It has been revealed that poor early development of insulin-producing β-cells causes type 2 diabetes later in life (3-7). Considerable attention is being devoted to the potential role of epigenetic modifications including DNA methylation in mediating the influence of environment on both type 1 and type 2 diabetes (25). It has been suggested that DNA methylation changes the expression of genes associated with various aspects of glucose metabolism and in particular glucose intolerance, β-cell proliferation and β-cell dysfunction, which result in diabetes mellitus (26). This gives rise to the speculation that maternal hyperglycemia may change the normal DNA methylation pattern of cell cycle inhibitory genes *CDKN2A* and *CDKN2B* in pancreatic islets of rat offspring. In the current paper, we demonstrated that possible links exist between fetal exposure to maternal GDM and DNA methylation of *CDKN2A/B* promoter in pancreatic islets.

The significance of maintaining appropriate β-cell growth for glucose homeostasis is evident (12). Recently, much effort has been directed to understanding the molecular mechanisms regulating β-cell proliferation. Previous research studies indicated that *CDK4-pRB-E2F1* pathway directly regulates β-cells proliferation (13). Furthermore, this pathway controls the expression of Kir6.2, a key factor involved in the regulation of insulin secretion (11, 13). Previous studies implicate CDK4 as a major regulator of pancreatic β-cells proliferation. An example is the study by Annicotte et al. (27), in which they found that clearance of glucose subsided in mice treated with a CDK4 inhibitor. In our recent study on a rat model, it was observed that GDM can significantly downregulate the *CDK4-pRB-E2F1* pathway in Langerhans islets of the offspring. *Our results* were thus consistent with earlier *studies mentioned above*, suggesting that there exists a link between CDK4 and the risk of diabetes (14). We hypothesized that inhibition of CDK4 kinase activity and subsequently inhibition of *CDK4-pRB-E2F1* pathway in GDM offspring may be caused by demethylation of *CDKN2A* and *CDKN2B* as CDK4 inhibitors. We therefore decided to study the association between GDM and DNA methylation of *CDKN2A/B* in pancreatic islets of rat offspring. Our findings demonstrated that CDKN2A promoters (161 to +281 bp) were hypomethylated in the OGD samples. Furthermore, there was a correlation between the increase in CDKN2A expression (in both mRNA and protein levels) and the promoter hypomethylation status. The difference in the average methylation levels for CDKN2A was signiﬁcant between the control and the OGD samples. On the other hand, some common methylation sites have seen in the control samples (CpG8 for *CDKN2A* and CpG9 for *CDKN2B*).

Such findings indicate that the differential methylation levels of these two CpG sites may be related to poor proliferative capacity of pancreatic β-cells. Human studies have validated the connection between the variants in *CDKN2A/B* and type 2 diabetes (28, 29). In line with previous studies, our data associates CDKN2A/B with the risk of diabetes in the offspring of mothers with gestational diabetes. Nonetheless, the procedure, through which the *CDKN2A/B* locus affects diabetes risk is yet to be discovered.

## Conclusion

The present research study provided for the first time, evidence that intrauterine exposure to hyperglycemia causes hypomethylation of *CDKN2A* and *CDKN2B* in pancreatic islets derived from the offspring of GDM rats. This differential methylation was most notable in CpG8 and CPG9 for *CDKN2A* and *CDKN2B*, respectively. These results suggest that a loss of methylation and overexpression of these cell cycle inhibitory genes possibly increase the susceptibility of type 2 diabetes through an inhibited *cyclin* *D1-CDK4* complex formation, leading to a decreased β-cell mass and mild hyperglycemia in the GDM-exposed offspring. Meanwhile, further investigations and larger-sclae studies are needed to completely investigate the molecular process of inducing diabetes in the offspring by GDM.
